# Bacterial community profiling of Malaysian drinking water reservoirs using metagenomic amplicon sequencing

**DOI:** 10.1186/s12863-026-01422-w

**Published:** 2026-04-10

**Authors:** Muhammad Farhan Roslan, Mohd Faiz Mat Saad, Suriyanti Su Nyun Pau, Syazwani Basir, Habsah Aziz, Muhamad Afiq Akbar, Hamidun Bunawan

**Affiliations:** 1 Institute of Systems Biology, Universiti Kebangsaan Malaysia, Bangi, Selangor 43600 Malaysia; 2 Department of Earth Sciences and Environment, Faculty of Science and Technology, Universiti Kebangsaan Malaysia, Bangi, Selangor 43600 Malaysia; 3 Department of Biological Sciences and Biotechnology, Faculty of Science and Technology, Universiti Kebangsaan Malaysia, Bangi, Selangor 43600 Malaysia; 4 Department of Microbiology, Faculty of Biotechnology and Biomolecular Sciences, Universiti Putra Malaysia, Serdang, Selangor 43400 Malaysia; 5 Environmental Health Risk Sector, Engineering Services Division, Ministry of Health Malaysia, Federal Government Administration Centre, Putrajaya, 62590 Malaysia

**Keywords:** 16 S rRNA gene amplicon, Freshwater reservoir, Bacterial community

## Abstract

**Objectives:**

Microbial communities in freshwater are pivotal for driving nutrient transformation, bioremediation, and maintaining the health balance of these ecosystems. However, microbial communities in freshwater ecosystems may undergo rapid shifts in composition in response to environmental changes. In some cases, these shifts may signal ecological imbalance. In the tropics like Malaysia, high humidity coupled with high temperatures and seasonal rainfalls creates an even hotter and highly variable environmental conditions that promote microbial proliferation and increase the risk of introducing potentially pathogenic microorganisms from surrounding anthropogenic sources via surface runoff. The current study offers a comprehensive characterisation of bacterial community composition in Malaysian freshwater drinking reservoirs using high throughput 16 S rRNA gene amplicon sequencing. This approach enables bacterial community profiling and supports initial microbial risk assessment in these vital freshwater ecosystems.

**Data description:**

State Authorities permitted the collection of water samples from eight freshwater reservoirs within Peninsular Malaysia’s protective zones. The 16 S rRNA gene’s V3-V4 hypervariable region was amplified for next generation sequencing. Raw DNA sequence data in FASTQ format were quality-filtered, adapter-trimmed and processed using a QIIME v1.9.1 based pipeline that integrated standard bioinformatics tools for OTU clustering and taxonomic assignment. Utilising R v3.3.1, statistical analyses and data visualisations were performed on this dataset. Characterising the community structure of bacteria in these important freshwater ecosystems is the first step to working with this dataset.

## Objective

In Malaysia, freshwater reservoirs are essential sources of drinking water catering to both urban and rural communities. However, these resources are increasingly affected by human activities such as agricultural practices and land conversion for aquaculture. Climate change further exacerbates these pressures and alters the composition and function of microbial communities in the water catchments that supply the reservoirs [1–5]. These pressures can disrupt the resident microbial communities and may increase the risk of pathogen proliferation with adverse consequences to water quality and public health [4, 6].

For efficient bioremediation and nutrient recycling in a freshwater system, orderly and functioning microbial community structures are crucial [7]. Disturbance to these microbial communities may indicate a potential imbalance in the system [8, 9]. Malaysia’s hot, humid tropical climate with prominent seasonal rainfall enhance surface runoff can stimulate the microbial growth and increase the risk of microbial contamination from anthropogenic sources in drinking water catchments [7, 9, 10]. Despite this, microbial diversity in Malaysian drinking water reservoirs remains poorly characterised.

Culture-dependent isolation methods capture only a small fraction of the total microbial diversity and do not fully resolve community wide structures and dynamics [11]. In contrast, large scale 16 S rRNA gene amplicon sequencing provides a more comprehensive, culture independent view of microbial community composition and diversity.

In this study, we characterised the microbial community composition of eight freshwater drinking reservoirs across Peninsular Malaysia using 16 S rRNA gene amplicon sequencing. The obtained results are expected to provide a baseline resource for microbial risk assessment in Malaysian drinking water and support future planning and decision making related to water quality management.

## Data description

Freshwater samples were collected from eight drinking water reservoirs located in different states of Malaysia, which serve as the main sources of drinking water for the local communities. The selected reservoirs capture different environmental and anthropogenic gradients. Each of the following locations was sampled for water across the country’s different geographical regions: reservoirs in Johor for the southern region - Bekok (2°06’13"N 103°04’40"E) and Sembrong (1°58’38"N 103°11’03"E) as well as Melaka’s Durian Tunggal (2°20’1’’N 102°18’14’’E) and Negeri Sembilan’s Talang (2°47’13’’N 102°8’53’’E) reservoirs, Selangor’s Labohan Dagang (2°47’52’’N 101°35’56’’E) reservoir, Pulau Pinang’s Air Itam (5°23’42’’N 100°15’47’’E) reservoir, Perlis’s (northmost region) Timah Tasoh (6°34’48’’N 100°13’21’’E) reservoir, and Chereh (4°02’29’’N 103°2’05’’E) reservoir in Pahang comprising the eastern region. To minimise variability caused by surface runoff events, sampling was carried out during the dry season. The data set reflects bacterial community composition at single time point and does not capture seasonal variation. Permissions were obtained from relevant State Authorities prior sampling activities.

At each designated sampling location, a total of 2 L of surface water was collected by grab sampling at approximately 0.3 m depth using a sterile Van Dorn bottle and aseptically transferred into sterile semi-transparent, high-density polyethylene (HDPE) bottles. For each reservoir, water was collected from three locations: near the water treatment plant intake, the central open water area and the site farthest from the intake. Samples were immediately stored at 4–10 °C and transported on ice to the laboratory facilities at Universiti Kebangsaan Malaysia on the day of collection. Upon arrival at the laboratory, samples collected from the three locations within each reservoir were pooled to provide a representative overview of the overall reservoir bacterial community rather than to enable site-specific comparisons within individual reservoirs. Then 1 L aliquot of the pooled sample was subsequently filtered through a sterile 0.2 μm pore-size nitrocellulose membrane filter (Whatman™) to concentrate microbial biomass [12, 13].

Environmental DNA (eDNA) was extracted from membrane retained microbial biomass using a modified cetyltrimethylammonium bromide (CTAB) protocol, adapted from the method described by Minas et al. [14]. The integrity of extracted eDNA was assessed via agarose gel electrophoresis (0.8% w/v). eDNA quantity and quality were determined using a NanoDrop™ spectrophotometer (Thermo Fisher Scientific, USA) to assess A260/280 and A260/A230 ratios, and a Qubit^®^ dsDNA HS Assay Kit (Thermo Fisher Scientific, USA) for precise quantification of eDNA concentration.

A MetaVX Library Preparation Kit was used to prepare the sequencing library. To generate amplicons that span the V3 and V4 hypervariable regions of the 16S rRNA gene of bacteria, 20–50 ng of eDNA was used. The amplification of the V3-V4 hypervariable region of the 16S rRNA gene was performed using the forward primer 5‘-CCTACGGRRBGCASCAGKVRVGAAT-3’ and the reverse primer 5‘-GGACTACNVGGGTWTCTAATCC-3’. Concentration was assessed with a microplate reader (Tecan, Infinite 200 Pro) and fragment size was assessed using 1.5% agarose gel electrophoresis, where the expected size was approximately 600 bp. The first-round PCR products were used as templates for the second-round amplicon enrichment PCR. In the same step, indexed adapters were attached to the 16 S rRNA amplicons, creating indexed libraries that were ready for sequencing on the Illumina MiSeq platform using 250 bp paired-end sequencing.

Quality control of the raw sequence reads in FASTQ format was performed using Cutadapt program v1.9.1 [15] which was used to trim adapter sequences and low-quality bases (Phred quality scores < 20 at the 5’ and 3’ ends) Chimeric sequence detection and removal were done using the UCHIME algorithm with the RDP Gold reference database. Non-Chimeric paired-end sequences were merged using Vsearch software v1.9.6 [16] and this produced 5,368,606 merged good quality sequences across all samples which were clustered into 5,559 OTUs at 97% sequence similarity. Taxonomic classification of OTUs was performed in a single pipeline using the RDP classifier v2.2 implemented in QIIME v1.9.1 and trained on the SILVA 138 reference database [17–19]. Alpha diversity was assessed using Shannon and Simpson diversity indices implemented in QIIME v1.9.1 to quantify community richness and evenness. Beta diversity describing community similarity and dissimilarity between samples was assessed using Bray-Curtis dissimilarity and visualised with the vegan package in R v3.3.1 [20]. Table 1 lists all deposited data files and data sets.

**Table 1:** Overview of data files and data sets

Label	Name of data file/data set	File types (file extension)	Data repository and identifier (DOI or accession number)
Data file 1	PCR products for 16 S rRNA V3-V4 amplicons from selected Malaysian drinking reservoirs	Image file (.jpeg)	10.6084/m9.figshare.30424846 [21]
Data file 2	Relative abundance of the 30 most abundant bacterial taxa in eight selected Malaysian drinking reservoirs	PDF file (.pdf)	10.6084/m9.figshare.30844064 [22]
Data file 3	Principal coordinates analysis (PCoA) of bacterial community composition in eight selected Malaysian drinking reservoirs	PDF file (.pdf)	10.6084/m9.figshare.30844118 [23]
Data file 4	Genus level alpha diversity and sequencing statistics for bacterial communities in eight selected Malaysian drinking reservoirs	PDF file (.pdf)	10.6084/m9.figshare.30845114 [24]
Data set 1	16 S rRNA gene amplicon sequencing of bacterial communities from Bekok Reservoir, Peninsular Malaysia	FASTQ files (.fastq)	https://identifiers.org/insdc.sra:SRR34653296 [25]
Data set 2	16 S rRNA gene amplicon sequencing of bacterial communities from Sembrong Reservoir, Peninsular Malaysia	FASTQ files (.fastq)	https://identifiers.org/insdc.sra:SRR34653295 [26]
Data set 3	16 S rRNA gene amplicon sequencing of bacterial communities from Durian Tunggal Reservoir, Peninsular Malaysia	FASTQ files (.fastq)	https://identifiers.org/insdc.sra:SRR34653294 [27]
Data set 4	16 S rRNA gene amplicon sequencing of bacterial communities from Talang Reservoir, Peninsular Malaysia	FASTQ files (.fastq)	https://identifiers.org/insdc.sra:SRR34653293 [28]
Data set 5	16 S rRNA gene amplicon sequencing of bacterial communities from Labohan Dagang Reservoir, Peninsular Malaysia	FASTQ files (.fastq)	https://identifiers.org/insdc.sra:SRR34653292 [29]
Data set 6	16 S rRNA gene amplicon sequencing of bacterial communities from Air Itam Reservoir, Peninsular Malaysia	FASTQ files (.fastq)	https://identifiers.org/insdc.sra:SRR34653291 [30]
Data set 7	16 S rRNA gene amplicon sequencing of bacterial communities from Timah Tasoh Reservoir, Peninsular Malaysia	FASTQ files (.fastq)	https://identifiers.org/insdc.sra:SRR34653290 [31]
Data set 8	16 S rRNA gene amplicon sequencing of bacterial communities from Chereh Reservoir, Peninsular Malaysia	FASTQ files (.fastq)	https://identifiers.org/insdc.sra:SRR34653289 [32]

## Limitations

Although 16 S rRNA gene amplicon sequencing was useful for determining the bacterial taxonomic composition within the freshwater microbiomes in these reservoirs, it provides only indirect insight into community functional potential. In principle, taxonomic profile from 16 S data can be combined with prediction tools based on ancestral state reconstruction, but these approaches are less precise than gene-level functional annotation from shotgun metagenomic.

Additionally, the sampling strategy based on aggregating water from three points within each reservoir to obtain a descriptive profile of the bacterial community, while useful, limited in-depth comparisons within each reservoir and across different time periods. Future studies incorporating shotgun metagenomics analysis are warranted to elucidate the functional roles of these microbial communities and to characterise the contributions of non-bacterial organisms. To gain a more comprehensive understanding of temporal dynamics, we recommend the implementation of longitudinal sampling strategies encompassing a full annual cycle, capturing variations associated with seasonal shifts in precipitation and temperature.

## Data Availability

The data described in this Data Note are freely available in the NCBI Sequence Read Archive under BioProject accession PRJNA1294531. Individual SRA Run accessions for each reservoir sample are listed in Table 1 and the BioProject record can be accessed at: https://www.ncbi.nlm.nih.gov/bioproject/PRJNA1294531. Processed data files underlying the diversity summaries and figures have been deposited in Figshare. These include the genus level relative abundance generates in Data file 2. The PCoA coordinates used for the beta diversity analysis in Data file 3 and the genus level alpha diversity and sequencing statistics table used in Data file 4. Data set names, file types and DOIs for all Figshare records are also provided in Table 1.

## Abbreviations

16 S rRNA: 16 S ribosomal RNA
CTAB: Cetyltrimethylammonium Bromide
DNA: Deoxyribonucleic acid
HDPE: High-Density Polyethylene
OTU: Operational taxonomic unit
PE: Paired end
RNase: Ribonuclease
